# 876 Effects of Antibiotic Resistance on Burn Outcomes

**DOI:** 10.1093/jbcr/iraf019.407

**Published:** 2025-04-01

**Authors:** Rashid Syed, Suhaib Shah, Abbas Karim, Zain Akbar, Farhad Marzook, Ridwan Syed, Juquan Song, Steven Wolf, George Golovko, Amina El Ayadi

**Affiliations:** University of Texas Medical Branch; University of Texas Medical Branch; University of Texas Medical Branch, John Sealy School of Medicine; University of Texas Medical Branch; University of Texas Medical Branch; University of Texas Medical Branch; University of Texas Medical Branch; University of Texas Medical Branch; University of Texas Medical Branch; University of Texas Medical Branch

## Abstract

**Introduction:**

Antibiotic resistance remains the main challenge in burn wound infections. With the emergence of multidrug-resistant bacteria, this study aims to measure the effect of antibiotic resistance on health outcomes, including sepsis, urinary tract infections, pneumonia, and mortality in burn patients.

**Methods:**

The TriNetX database, a large, federated research network containing patient-de-identified data, was utilized to identify burn patients and compare those who developed antibiotic resistance post-burn to those who did not. Patient cohorts were stratified based on % TBSA burn, infection with gram-positive bacteria, or infection with gram-negative bacteria. Cohorts were propensity-matched based on age at index, gender, race, ethnicity, and percentage of total body surface area (%TBSA) burned. Risk analysis and measures of association were determined using the analytical tools within the TrinetX database with a significance level of p< 0.05. The occurrence of urinary tract infections (UTI), pneumonia, sepsis, and death within three months of burn injury was compared between patient cohorts with and without antibiotic resistance.

**Results:**

Data analysis was performed using data from eight patient cohorts that were propensity-matched; 1-All TBSA burn ± Antibiotic resistance, 2- Severe burn patients (>20% TBSA) ± Antibiotic resistance, 3- All TBSA with Gram-positive infection± Antibiotic resistance; 4- All TBSA with Gram-negative infection± Antibiotic resistance. Our data show that male burn patients were disproportionately affected across all antibiotic-resistance cohorts (52.68%, 64.51%, 62.05%, and 64.62% respectively). Antibiotic resistance increased the risk of sepsis by 2.6 in patients with greater than 20% TBSA burn. Antibiotic resistance in Pseudomonas-infected patients increased the risk of developing urinary tract infections by 2.3 times. The risk of Pneumonia was increased in all burn cohorts by 1.7 irrespective of the type of infection or burn size. Antibiotic resistance in patients with gram-positive infections experienced the highest mortality rates, with 1.6 times increased risk compared to those with no antibiotic resistance. Antibiotic resistance did not increase the risk of mortality in patients with severe burns infected with Gram-negative bacteria.

**Conclusions:**

Burn patients with antibiotic resistance experience significantly worse outcomes, including higher rates of sepsis, urinary tract infections, pneumonia, and mortality.

**Applicability of Research to Practice:**

This study demonstrated that antibiotic resistance is associated with worse outcomes, such as elevated rates of sepsis, urinary tract infections, pneumonia, and mortality. These findings may guide healthcare providers to improve antibiotic use by identifying at-risk patients early and selecting the appropriate antibiotics to combat resistance.

**Funding for the Study:**

Database funding by National Center for Advancing Translational Sciences.

